# Changes and Trends—Efficiency of Physical Blowing Agents in Polyurethane Foam Materials

**DOI:** 10.3390/ma16083186

**Published:** 2023-04-18

**Authors:** Haozhen Wang, Xiong Yang, Yingshu Liu, Lin Lin

**Affiliations:** School of Energy and Environmental Engineering, University of Science and Technology Beijing, Beijing 100083, China

**Keywords:** polyurethane, physical blowing agent, efficiency, equilibrium, environmental friendliness

## Abstract

This work developed a novel method for measuring the effective rate of a PBA (physical blowing agent) and solved the problem that the effective rate of a PBA could not be directly measured or calculated in previous studies. The results show that the effectiveness of different PBAs under the same experimental conditions varied widely, from approximately 50% to almost 90%. In this study, the overall average effective rates of the PBAs HFC-245fa, HFO-1336mzzZ, HFC-365mfc, HFCO-1233zd(E), and HCFC-141b are in descending order. In all experimental groups, the relationship between the effective rate of the PBA, $r_{ePBA}$, and the initial mass ratio of the PBA to other blending materials in the polyurethane rigid foam, w, demonstrated a trend of first decreasing and then gradually stabilizing or slightly increasing. This trend is caused by the interaction of PBA molecules among themselves and with other component molecules in the foamed material and the temperature of the foaming system. In general, the influence of system temperature dominated when w was less than 9.05 wt%, and the interaction of PBA molecules among themselves and with other component molecules in the foamed material dominated when w was greater than 9.05 wt%. The effective rate of the PBA is also related to the states of gasification and condensation when they reach equilibrium. The properties of the PBA itself determine the overall efficiency, while the balance between the gasification and condensation processes of the PBA further leads to a regular change in efficiency with respect to w around the overall average level.

## 1. Introduction

Polyurethane rigid foam (PURF) is widely used in white appliances, cold storage, construction, and other fields due to its excellent thermal insulation performance [1,2,3]. PURF is usually prepared by vigorously mixing two reactants, i.e., polyols and polymerized MDI, etc. [4], to form a rigid, foam-like polymer after the curing reaction. PURF can use water as chemical blowing agent (CBA) for foaming or a physical blowing agent (PBA) to promote foaming [5]. A PBA is usually a liquid with a low boiling point that evaporates and vaporizes when the reaction medium heats up due to the exothermic heat of the reaction. HFC-245fa, HCFC-141b, HFC-365mfc, HFO-1336mzz(Z) and HFCO-1233zd(E) are currently widely used PBAs in the polyurethane foam field. These blowing agents are classified as second-, third-, and fourth-generation blowing agents. The fourth-generation blowing agent is a completely environmentally friendly PBA, with an ozone depletion potential (ODP) [6] of 0 and a very low global warming potential (GWP) [7]. Table 1 provides a description of the relevant properties for each generation of the PBA system [8,9,10,11].

In polyurethane foam systems, the PBA and water usually work together to produce foam. The foaming process of polyurethane foam systems involves a series of steps that transform a low-viscosity liquid into a high-viscosity gel and eventually into a solid foam. During this process, any variations in the PBA can have a significant impact on the structure, properties, and production cost of the foam. In the polyurethane foaming process, the PBA has two typical states: the vaporized state and the dissolved state. Between the two states of the PBA, its state of effective gasification foaming is the first to pay attention to. This state is directly related to the molded density of the polyurethane foam and indirectly affects the thermal conductivity, compressive strength, sound absorption, and dimensional stability, etc. As the polyurethane foaming process is very complex, including mass transfer, heat transfer, gasification, condensation, and other processes, traditional research has mostly been performed from the perspective of a foaming simulation calculation. Rojas et al. [12] considered the foaming kinetics as controlled by the rate of heat generation and studied the free-rising, R-11 rigid polyurethane foam for the first time. The basic theoretical ideas of the Rojas study were cited and improved by many subsequent researchers. Baser et al. [13,14] improved the Rojas model and considered the dilution effect of the blowing agent on the reaction mixture. Tesser et al. [15] further optimized a model to include heat transfer and modified the vapor–liquid equilibrium description of the blowing agent and polymer phases using an extended Flory–Huggins equation. Harikrishnan and Khakha [16] studied the kinetic simulation of a polyurethane foam reaction and film thinning, focusing on the cell wall thinning during foam formation and the cell opening in soft polyurethane foam molding. Suppes et al. [17] studied a simulation of urethane reaction processes and foaming processes for an analytical method and formulation development as a means of predicting the role of alternative polyols in a formula. Zhao et al. [18] first reported that the method of solving ordinary differential equations using the MATLAB program was used to establish a theoretical model to simulate the foaming process of a PURF. Gandhi et al. [19] developed a model that combined equations of energy balance, the kinetics of isocyanate’s reactions with water and polyol, and the nucleation and growth of CO_2_ bubbles to predict the bubble size and distribution of polyurethane foams blown using water as a chemical blowing agent. Modesti et al. [20] developed a theoretical model to predict the temperature profile, foam density, and other physico-mechanical properties using oligomeric isocyanate and a mixture of polyether polyols blown by either water, methyl formate, or n-pentane. Raimbault et al. [21] developed an analytical model for polyurethane foams based on FOAMAT ^®^ experiments; this model was verified using finite element software.

In recent years, some researchers have begun to pay attention to the efficiency of blowing agents. Among these researchers were Baser et al. [13,14], who noticed the low efficiency of the CFC-11 foaming agent when simulating polyurethane foaming dynamics; however, they did not carry out further research. Moameri et al. [22] used a MATLAB simulation code to simulate the kinetics and physical properties of the polyurethane foaming process when the efficiency of water in their experimental polyurethane formulation was set to more than 85% and the efficiency of n-pentane was set to 16–30%. However, they did not investigate the low efficiency of the blowing agent. Shen et al. [23] studied the physical process of polyurethane box foaming and quantitatively modeled the foam density. They found that in their study, the final density of the foam was only 30–90% of the predicted density. While the problem of the low efficiency of the blowing agent has been identified, it has not yet been thoroughly researched or received adequate attention.

It can be seen that in previous studies, the problem of PBA efficiency in PURF has been addressed by many researchers. However, the PBA efficiency cannot be directly measured or calculated based on the relevant calculation using dynamic modeling. A common practice in such studies is to pre-set the PBA activity coefficient or efficiency and then modify it using the actual test data. Due to the simplification of the modeling equation itself and the influence of water as a CBA in the foaming formula, the calculated PBA efficiency often deviates from the actual situation. At present, there are currently no systematic studies of the PBA efficiency of the second-, third-, and fourth-generation blowing agents HFC-245fa, HCFC-141b, HFC-365mfc, HFO-1336mzz(Z), and HFCO-1233zd(E).

This study differs from the previous work in four ways: (a) a complete methodology for measuring PBA efficiency has been developed; (b) the properties of the PBA after gasification as a real gas rather than an ideal gas were considered in the study; (c) the effectiveness of the second-, third-, and fourth-generation blowing agents HCFC-141b, HFC-365mfc, HFC-245fa, HFO-1336mzzZ, and HFCO-1233zd(E) was tested using this method; (d) the variation in the PBA effective rate with the PBA content and its influencing factors were systematically analyzed. The results of this study can allow users to control the efficiency of PBAs when using them, thus improving the environmental protection of the polyurethane foaming process, and can provide more targeted and cost-effective options for different users to choose PBAs.

## 2. Modeling Methods

During the foaming process, the mass of the PBA gasified and retained in the foam cells is the effective mass of the PBA, and the efficiency of the PBA can be expressed as the ratio of the effective PBA mass to the total PBA mass added to the material. The total mass of the PBA can be obtained by accurately weighing the relevant material components, and the effective mass of the PBA is difficult to obtain; there is no relevant report in previous studies. In this study, taking the part of the foam volume increase during foaming process as the core, the effective mass of the PBA during the foaming process was calculated by combining the limited measurable physical parameters with relevant formulas. The volume of the PURF can be seen as the sum of the volume occupied by the PU skeleton and various blowing agent gases. As a solid, the density change in the PU skeleton is minimal; thus, its volume can be considered equivalent to the volume of the polyurethane mixture. The total blowing agent gas in the PURF contains vaporized PBA and CO_2_ generated by the reaction of water with NCO. The effective PBA volume is actually the volume of the vaporized PBA in the PURF, excluding the PU backbone and CO_2_. The effective mass of the PBA is actually the mass of the PBA that produces the volume of the effective PBA, and the effective rate of the PBA is the ratio of the effective mass of the PBA to the total mass of the PBA added to the material. As a gas, the state of the PBA can be expressed by its related gas state equation. After measuring the two key physical quantities of pressure and temperature in the state equation, the volume of the gaseous PBA can be determined. This study further considers the compression coefficient of the gaseous PBA as a real gas, which can express the volume occupied by the gaseous PBA more accurately. The specific method and model for calculating the effective mass of the PBA are shown as follows.

### 2.1. The Actual Increased Volume, $V_{P}$, of Foam

Due to the irregular growth shape of PURF in the foaming process and some surface defects, it is difficult to accurately measure its volume using the traditional measurement method. This problem can be solved by using the method of adding water to replace the difference [8].

**Figure 1:**
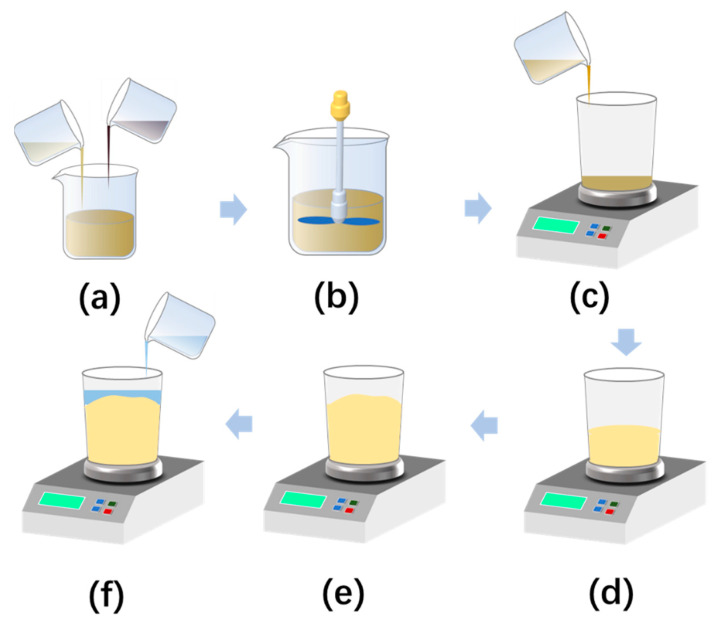
The method of adding water to compensate for the difference is described as follows: (**a**) add A-side and B-side; (**b**) mix and stir; (**c**) transfer the mixture into a large beaker for foaming; (**d**) perform PURF foaming; (**e**) complete the foam molding process; (**f**) add water to fill any voids.

The method of adding water to replace the difference is shown in Figure 1. This approach takes advantage of water’s extremely low viscosity and excellent flowability to prevent the numerous defects that can occur during the PURF foaming process. Moreover, it exhibits a remarkable tolerance for errors in the foam produced using various foaming methods. Additionally, this technique can be utilized to determine the volume of irregular and solid objects that have imperfections.

The real volume of PURF can be calculated using Equation (1):

(1) $$V_{P}=\frac{m_{t}-m_{a}+m_{f}}{\rho_{w}}-\frac{m_{b}}{\rho_{m}}$$

To determine the actual volume of the foam, the standard volume, $V_{c}$ (cm^3^), of the beaker was initially established and measured, enabling the true volume at the water addition scale to be determined. The mass of the water added to the empty beaker at the scale mark was denoted as $m_{t}$ (g). Once foam growth and solidification were completed, the total mass, $m_{f}$ (g), of the system was weighed. At the temperature of $T_{w}$, the water was added to the mark, and the mass of the water, $m_{a}$, was read at the scale mark on the beaker. Finally, Equation (1) was employed to calculate the actual increased volume, $V_{P}$, (cm^3^) of the foam using the aforementioned variables and the density of the PU materials, $\rho_{m}$ (g·cm^−3^). In addition, the physical quantities to be measured are the material total mass for the start of foaming, $m_{b}$ (g), and the density of the water (at water temperature $T_{w}$), $\rho_{w}$ (g·cm^−3^).

### 2.2. Measure the Density of Polyurethane Mixture

Since the polyurethane’s A-side and B-side react quickly after mixing, and as a large number of bubbles will be introduced during the stirring process, the measurement of the density of the polyurethane mixture is further affected. The densities of the A-side and B-side can be measured separately, and the contribution of the intermolecular forces between the A-side and B-side molecules is neglected in the calculation. The total volume of the mixture of the A-side and B-side is assumed to be the sum of their individual volumes, and the density of the polyurethane mixture $\rho_{m}$ (g·cm^−3^) is calculated using Equation (2).

(2) $$\rho_{m}=\frac{m_{B}+m_{A}}{V_{B}+V_{A}}=\frac{R_{AB}m_{A}+m_{A}}{\frac{R_{AB}m_{A}}{\rho_{B}}+\frac{m_{A}}{\rho_{A}}}=\frac{R_{AB}+1}{\frac{R_{AB}}{\rho_{B}}+\frac{1}{\rho_{A}}}$$

where $R_{AB}$ is the mass ratio of the B-side to the A-side, $R_{AB}=\frac{m_{B}}{m_{A}}$, $\rho_{A}$ (g·cm^−3^) is the density of the A-side, $m_{A}$ (g) is the mass of the A-side used, $\rho_{B}$ (g·cm^−3^) is the density of the B-side, $m_{B}$ (g) is the mass of the B-side used, $V_{A}$ (cm^3^) is the volume of the A-side, and $V_{B}$ (cm^3^) is the volume of the B-side.

### 2.3. Measurement of Foam Pressure and Temperature during Polyurethane Foaming

**Figure 2:**
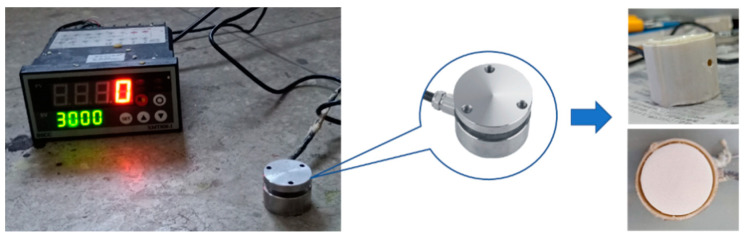
Pressure sensor with heat preservation.

**Figure 3:**
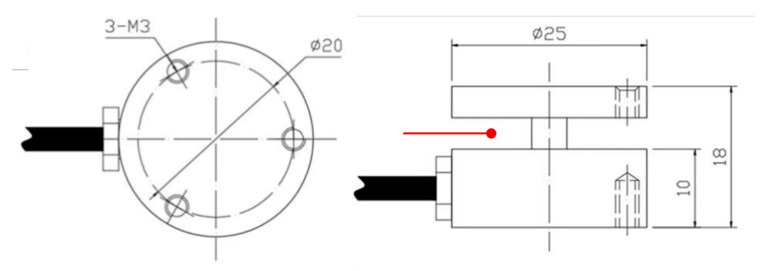
The size of pressure sensor. The red point is the thermocouple temperature measurement point.

During the PURF foaming process, as the reaction continues, the average molecular weight of the polymer continues to increase, and the viscosity inside the foam also increases. When the viscosity increases to a certain level, there will be a significant measurable pressure rise inside the foam, which can be measured using a pressure sensor. As the pressure sensor itself is made of metal, in order to reduce the impact of heat transfer from the foam, the pressure sensor itself must be covered with a hard insulation layer. The pressure sensor is shown before and after treatment in Figure 2. Due to the strong fluidity of the foam body in the foaming process and in order to ensure the normal operation of the pressure sensor, after the overall insulation treatment of the pressure sensor, it was also necessary to cover the outside of the processed sensor with a thin plastic film to prevent the penetration of polyurethane materials from affecting the test results. In the actual measurement process, the air circulation between the pressure sensor and the foam was isolated after the insulation and film wrapping treatment; therefore, the effect of the temperature increase inside the pressure sensor on the pressure of the moving component of the sensor also needed to be calculated together to further eliminate the error. The dimensions of the pressure sensor used in the study are shown in Figure 3. The temperature measurement inside the pressure sensor was carried out using a T-type thermocouple, and the measurement position was set between the moving and fixed components, as shown in Figure 3.

The reaction of active hydrogen atoms with isocyanate groups is an exothermic reaction. As the reaction progresses, the temperature inside the polyurethane foam will continue to increase. In the process of foam expansion, the spatial distribution of the foam temperature will show regular changes, which Wang et al. [24] systematically studied. The results showed that the temperature inside the foam rises uniformly, and as the foam temperature approaches the boundary, it gradually approaches the outside temperature (room temperature or mold temperature). In this study, since the dimensions near the boundary are much smaller relative to the diameter of the reaction vessel, the foam can be treated as a whole with a uniform internal temperature. A T-type thermocouple was used for the temperature measurement, and the temperature measurement point was set in the center of the foaming vessel. The foam pressure and temperature were measured and recorded when the foam stopped expanding.

### 2.4. Correction as Real Gas

The equation of state for an ideal gas is valid under the following conditions: the volume of the gas molecules themselves is ignored, and the molecules are considered geometric points with mass; it is assumed that there is no mutual attraction and repulsion between molecules, that is, regardless of molecular potential energy, the collision between the molecules and the wall is completely elastic and does not cause a loss of kinetic energy. A real gas differs from an ideal gas in the above aspects. Only at a high temperature and low pressure is a real gas infinitely close to an ideal gas.

During the PURF foaming process, the foam gradually expands under the combined action of the CO_2_ generated by the reaction and the heated and vaporized PBA. In this study, all gases were treated as real gases to further improve the accuracy of the study. Since most PBAs are polar substances, we used the Peng–Robinson (PR) equations with a high precision to calculate the state of the real gases. The PR equations are shown in Equations (3)–(6) [25].

(3) $$P=\frac{RT}{V_{n}-b}-\frac{a(T)}{V_{n}(V_{n}+b)+b(V_{n}-b)}$$

(4) $$a(T)=0.45724\frac{R^{2}T_{c}^{2}}{P_{c}}\alpha (T)$$

(5) $$b=0.07780\frac{RT_{c}}{P_{c}}$$

(6) $$\alpha (T)=[1+(0.37464+1.54226\omega -0.26992\omega^{2})(1-T_{r}^{0.5}){]}^{2}$$

where P (Pa) is the pressure, T (K) is the temperature, $V_{n}$ (m^3^·mol^−1^) is the molar volume of a real gas, $T_{c}$ (K) is the critical temperature, R = 8.314 J·mol^−1^·K^−1^ is the gas constant [26], $P_{c}$ (Pa) is the critical pressure, ω is the acentric factor, and $T_{r}$ is the contrast temperature, $T_{r}=\frac{T_{F}}{T_{c}}$.

In the PR equations, the parameter b represents the volume occupied by the molecules of the fluid being studied, and it is a function of temperature. The parameter b is given by Equation (5) and is related to the critical properties of the fluid. The parameter α(T) represents the strength of the intermolecular forces of the fluid being studied and is given by Equation (6). The value of α(T) is determined by the acentric factor ω and the temperature of the fluid. The acentric factor is a measure of how different a fluid’s behavior is from that of an ideal gas. The value of α(T) varies with temperature as the intermolecular forces become weaker at higher temperatures.

When using the PR equation to calculate CO_2_ and the PBA as the relevant properties of real gases, in the formula, P (Pa) takes the value $P_{F}$ (Pa), which is the pressure inside the foam when it stops expanding, and T (K) takes the value $T_{F}$ (K), which is the temperature inside the foam when it stops expanding.

As a cubic equation, it is difficult to solve the PR equation directly. However, in practice, the iterative method is usually used to solve it iteratively. Iteration can be easily performed using MATLAB or Python programming, and the criterion set to determine the convergence of the results is the difference between the $V_{n}$ values obtained by the nth and the n+1th iteration <10^−8^.

For any PBA as a real gas, the compression factor Z can be given by Equation (7):

(7) $$Z=\frac{P_{F}V_{n}}{RT_{F}}$$

The compression factor Z is the degree of deviation of the real gas from the ideal gas, and the measured $P_{F}$ and $T_{F}$ can be brought into Equations (3)–(6) and iteratively calculated to obtain the corresponding $V_{n}$ of the real gas. The compression factor Z of the PBA as a real gas can be calculated from Equation (7).

### 2.5. Removal of the Effect of CO_2_ Produced by the Water Reaction on Foam Expansion

Water is highly reactive with isocyanate. The CO_2_ produced by the reaction is conducive to nucleation and foam expansion in the early stage of foaming. Water is an important auxiliary blowing agent in the PURF foaming process, and the reaction process between the water and isocyanate is shown in Equation (8) [27]. In this study, a PBA blank group was set up, and the contribution of the CO_2_ generated by the reaction of the water with NCO was measured separately. The calculation results are used to remove the contribution value of CO_2_ to the foam expansion.

2 R−NCO + H_2_O → R−NHCONH−R + CO_2_ ↑ (8)

From Equation (8), 1 mol of water reacts with isocyanate to form 1 mol CO_2_. The actual increased volume of the foam, $V_{P}$, the pressure inside the foam when it stops expanding, $P_{F}$, and the temperature inside the foam when it stops expanding, $T_{F}$, were calculated experimentally by the PBA blank group. The compression coefficient of the CO_2_, $Z_{{CO}_{2}}$, was calculated by the PR equation.

The number of moles of effective water per unit mass at the start of foaming without PBA, $a_{H_{2}O}$ (mol·g^−1^), can be obtained by taking the total mass at the start of foaming, $m_{c_{0}}$ (g),the actual increased volume, $V_{P_{0}}$ (cm^3^), in the PBA blank group, and the above parameters in Equation (9).

(9) $$a_{H_{2}O}=\frac{P_{F}\;V_{P_{0}}}{Z_{{CO}_{2}}\;RT_{F}\;m_{c_{0}}}$$

### 2.6. Calculation of the Effective Rate of PBA

After obtaining the $P_{F}$ and $T_{F}$ data and calculating the $Z_{PBA}$, the part of the volume contributed by the PBA, $V_{PBA}$ (cm^3^), can be calculated using Equation (13). The number of moles of the effective PBA, $n_{ePBA}$ (mol), can then be further calculated. Due to the reaction of water and isocyanate during the foaming process, the volume of the foam is inflated by the combination of the CO_2_ generated from the reaction and the gasified PBA. Therefore, $V_{PBA}$ (cm^3^) is the difference between the volume of foam that is actually increased, $V_{P}$ (cm^3^), and the volume of CO_2_ occupied, as shown in Equation (12). Equation (10) represents the number of moles of effective water in the material total mass at the start of foaming, $n_{aH_{2}O}$ (mol), calculated from $a_{H_{2}O}$ (mol·g^−1^). With the calculated $n_{aH_{2}O}$ and $Z_{{CO}_{2}}$ and the measured $P_{F}$ and $T_{F}$ data, the part of the volume contributed by the CO_2_ in the foam, $V_{{CO}_{2}}$ (cm^3^), can be calculated by Equation (11).

The effective rate of the PBA relative to the total moles of PBA used, $r_{ePBA}$ (%), is defined by Equation (15). The effective rate of the PBA relative to the total mass of materials used, $r_{eT}$ (wt%), is defined by Equation (16), where $m_{c}$ (g) is the material total mass at the start of foaming without the PBA, $m_{ePBA}$ (g) is the mass of the effective PBA, $M_{PBA}$ (g·mol^−1^) is the molar mass of the PBA, and $Z_{PBA}$ is the compression coefficient of the PBA at the corresponding temperature and pressure.

(10) $$n_{aH_{2}O}=m_{c}\;a_{H_{2}O}$$

(11) $$V_{{CO}_{2}}=\frac{Z_{{CO}_{2}}RT_{F}}{n_{{aH}_{2}O}P_{F}}$$

(12) $$V_{PBA}=V_{P}-V_{{CO}_{2}}$$

(13) $$n_{ePBA}=\frac{P_{F}\;V_{PBA}}{Z_{PBA}RT_{F}}$$

(14) $$m_{ePBA}=n_{ePBA}\;M_{PBA}$$

(15) $$r_{ePBA}=\frac{n_{ePBA}}{n_{PBA}}$$

(16) $$r_{eT}=\frac{m_{ePBA}}{m_{c}}$$

## 3. Experimental

### 3.1. Raw Materials and Experimental Apparatus

PM-200, a polymeric diphenylmethane diisocyanate from Wanhua Chemical Group Co., Ltd. (Yantai, China), was employed to manufacture the foams. The characteristics of the isocyanate were a functionality of 2.7, an NCO content of 31%, and a viscosity of 180–250 mPa·s at 25 °C. R4110A, a high-functionality polyether polyol from Baichuan C.C.L. (Rugao, China), was used to manufacture the foams under study. The characteristics of the polyol used were a functionality of 4.3 and 440 mg KOH/g of hydroxyl. DMCHA from Intech Co., Ltd. (Beijing, China), was employed as a blowing catalyst, while PC41 and DBTDL from Intech Co., Ltd., were employed as gelling catalysts. BL-8950 from Menhover C.C.L. (Shanghai, China) was employed as a surfactant. The chemical blowing agent used was distilled water. TCPP from Yoke T.C.L (Wuxi, China). was employed as a flame retardant. The five PBAs were separately provided by the following companies: HCFC-141b was supplied by San’aifu Fluorine C.C.L. (Changshu, China), HFC-245fa and HFCO-1233zd(E) were supplied by Honeywell Co., Ltd. (New Jersey, USA), HFO-1336mzzZ was supplied by Chemours C.C.L. (Wilmington, DE, USA), and HFC-365mfc was supplied by Solvay Co., Ltd. (Brussels, Belgium).

JFS1100-ST, a high-speed frequency conversion dispersing machine, was supplied by Lichen Instrument T.C.L. (Shaoxing, China). An electronic balance, JJ2000B, was supplied by Shuangjie Electronic C.C.L. (Dongguan, China). A miniature plane capsule pressure sensor, JHHM-H3, and a pressure sensor display instrument, JH-808, were supplied by Jinnuo Sensor Co., Ltd. (Bengbu, China). A thermocouple temperature-measuring wire, TT-T-30, and a high-precision portable thermocouple thermometer, YET-640, were supplied by Suma Electric Instrument Co., Ltd. (Xinghua, China).

### 3.2. Experimental Procedure

The experimental results of the effectiveness of various PBAs in the PURF foaming process were analyzed and studied, with the foaming recipes for Group 1 and Group 2 reported in Table 2 and Table 3, respectively. Group 1 is the PBA blank group and Group 2 is the experimental group.

At room temperature, the proportions in Table 1 were followed to successively add and thoroughly mix each component of the A-side. Subsequently, an appropriate amount of A-side and a corresponding proportion of B-side were mixed together in a beaker, which was stirred at 2500 rpm for 10 s before being quickly poured into a plastic beaker. During and after foaming, the value required was recorded and the mass of the water added was measured. The previous mixed foaming operation was repeated, and the pressure sensor and thermocouple were used to measure the pressure and temperature when the foam stopped expanding.

## 4. Results and Discussion

### 4.1. Measurement of the Density of the Polyurethane Mixture

The initial mass ratio of the PBA to the other mixture materials in the PURF is w (wt%), and w is defined by Equation (17), where $m_{PBA}$ (g) is the initial PBA mass.

(17) $$w=\frac{m_{PBA}}{m_{c}}\times 100\%$$

**Figure 4:**
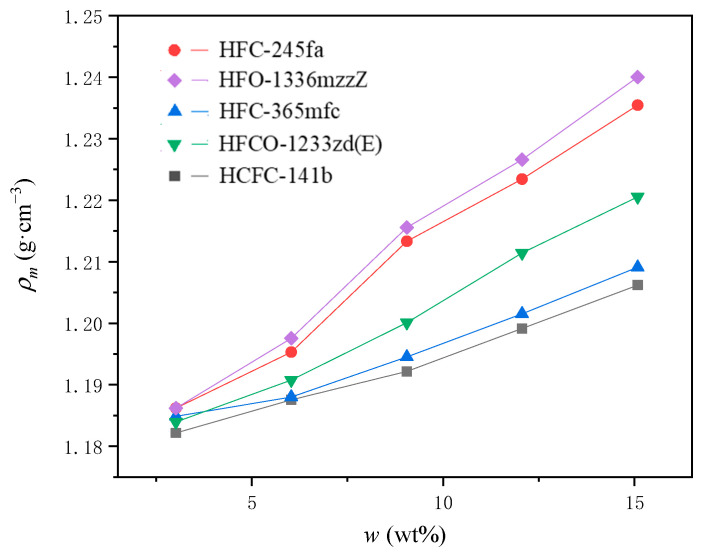
The relationship of the density of polyurethane mixtures with different PBAs, $\rho_{m}$, and the initial mass ratio of the PBA to the other blending materials in the PURF, w.

The density of polyurethane mixtures with different PBAs, $\rho_{m}$, can be calculated using Equation (2), and the relationship between $\rho_{m}$ and w is shown in Figure 4.

As the density of the PBA is generally higher than those of the other raw materials in the A-side, the $\rho_{m}$ therefore also increases when increasing the PBA addition. PBAs with higher densities cause the mixtures to be denser.

### 4.2. Measurement of the Internal Pressure of the Foam When It Stops Expanding during Polyurethane Foaming

**Figure 5:**
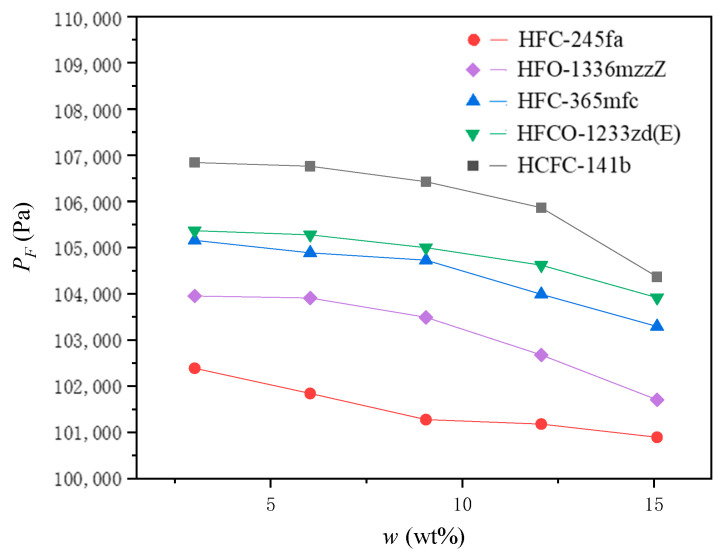
The relationship between the internal pressure of the foam when it stops expanding during polyurethane foaming, $P_{F}$, and the initial mass ratio of the PBA to the other blending materials in the PURF, w.

The relationship between the internal pressure of the foam when it stops expanding during polyurethane foaming, $P_{F}$, and the initial mass ratio of the PBA to the other blending materials in the PURF, w, is shown in Figure 5.

The foaming pressure of polyurethane systems with different PBA components showed a consistent, regular change, and the foaming pressure showed a decreasing trend with the increasing addition of the PBA. With the increase in the addition of the PBA, the heat absorbed by PBA gasification during foaming gradually increased, and the absorbed heat helped to delay the rise rate of the temperature inside the polyurethane foam and further delayed the formation of polyurethane gel, causing the foam to have a longer growth time. At the same time, as the amount of the PBA added increased, the mass of PBA vaporized per unit time in the foam increased accordingly, making the foam more likely to expand and grow faster. Both factors reduce the viscous resistance during foam growth and increase the fluidity of the foam, leading to a decrease in foaming pressure when the foam stops growing. Relatively speaking, when the PBA content is low, its effect on the reduction of the foaming pressure is limited, but when the PBA content reaches a certain level, its effect on the reduction of the foaming pressure is significantly accelerated.

With the exception of HFC-245fa, other PBA systems showed that the foaming pressure decreased slowly at first and then decreased more rapidly with the increasing PBA content in the system. HFC-245fa showed that beginning with a large reduction, the pressure then tended to stabilize gradually. The possible reason for this is that the foaming pressure of the HFC-245fa blown system itself was very low, and the overall fluidity of the system was significantly better than that of the other PBA systems. In addition, because the foaming fluidity was already very good and it is difficult to increase it further by increasing the HFC-245fa content, its foaming pressure tends to be essentially fixed.

Based on the pressure curve of each PBA, it can be seen that the pressure difference corresponding to different PBAs at the same PBA content is significant, which shows that the physical properties of the PBA itself have a much greater impact on the fluidity of the polyurethane foam than other factors. Among the five blowing agents tested, HFC-245fa has the most prominent performance in this regard, while HCFC-141b has the worst performance.

### 4.3. Measurement of the Internal Temperature of the Foam When It Stops Expanding during Polyurethane Foaming

**Figure 6:**
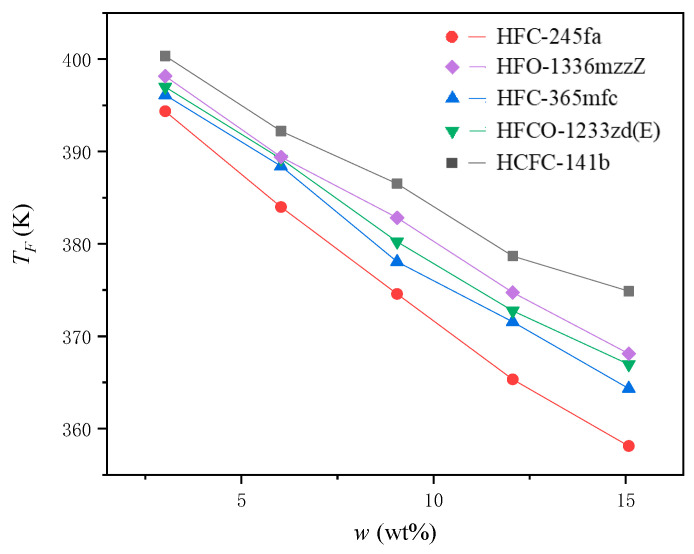
The relationship between the internal temperature of the foam when it stops expanding during polyurethane foaming, $T_{F}$, and the initial mass ratio of the PBA to the other blending materials in the PURF, w.

The relationship between the internal temperature of the foam when it stops expanding during polyurethane foaming, $T_{F}$, and the initial mass ratio of the PBA to the other blending materials in the PURF, w, is shown in Figure 6.

When the PURF growth stopped, the corresponding temperature of the polyurethane systems with different PBAs showed a constant and regular change. The foaming temperature showed a decreasing trend with the increasing PBA content. The decreasing slope of different PBAs was significantly different; this was related to the ability and the effective rate of the PBA to vaporize and absorb heat in the polyurethane systems.

### 4.4. Compressibility Coefficient of Various PBAs as Real Gases

**Figure 7:**
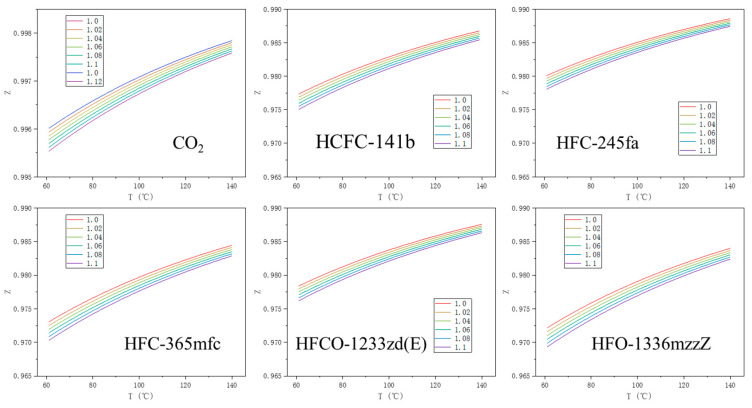
The compression factor of CO_2_, Z, and different PBAs as real gases at different pressures and temperatures. The different colored curves in the figure represent the value of the compression factor, Z, with the temperature at different pressures. The unit of the number in the legend data frame in the figure is 10^5^ Pa.

By iteratively calculating the PR equation and combining it with Equation (7), the compression factor Z of CO_2_ and various PBAs as real gases at different pressures and temperatures can be obtained. The compression factor of the gas molecules is plotted as a cluster curve, and the compression factor Z under the desired $P_{F}$ and $T_{F}$ can be determined by selecting the corresponding pressure and temperature values. The variation of the CO_2_ and the various PBA compression factors with pressure and temperature is shown in Figure 7.

### 4.5. Removal of the Effect of CO_2_ Produced by the Water Reaction on Foam Expansion

After the experiment on the PBA blank group, the corresponding blank group data were obtained in which the compression coefficient of CO_2_, $Z_{{CO}_{2}}$, was 0.998. The number of moles of the effective water per unit mass at the start of foaming without the PBA, $a_{H_{2}O}$, was 1.13 × 10^−4^ mol·g^−1^.

### 4.6. The Effective Rate of PBA

**Figure 8:**
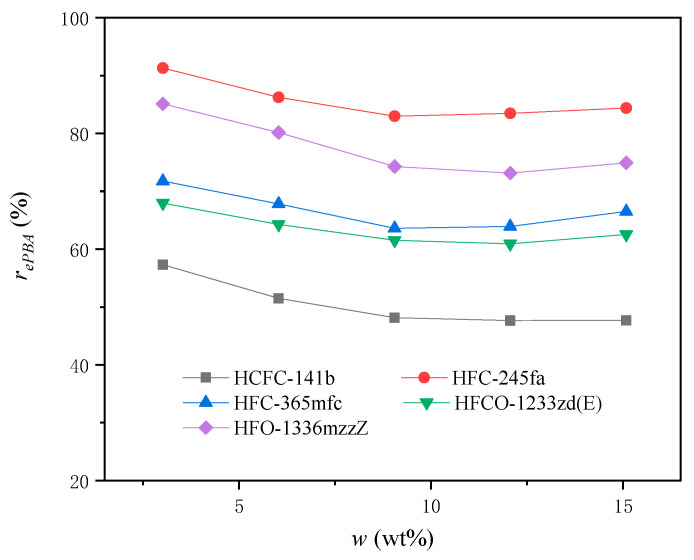
The relationship of the effective rate of PBA relative to the total moles of PBA used, $r_{ePBA}$, and the initial mass ratio of PBA to other blending materials in PURF, w.

**Figure 9:**
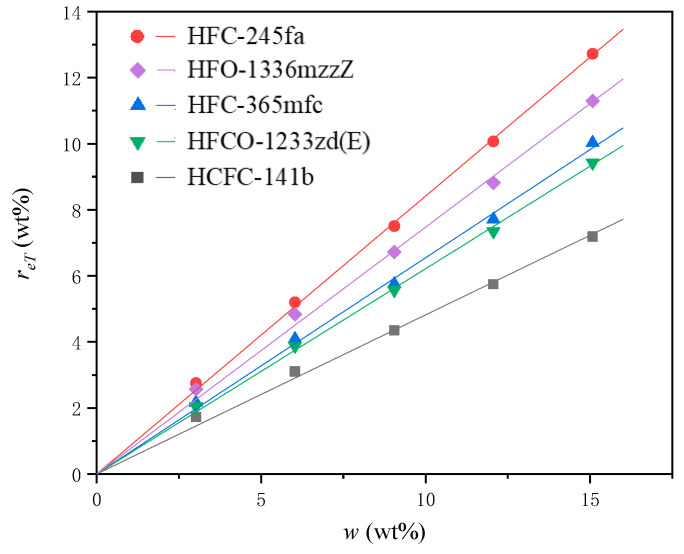
The relationship of the effective rate of PBA relative to the total mass of materials used, $r_{eT}$, and the initial mass ratio of PBA to other blending materials in PURF, w.

We can calculate the effective rate of the PBA in each experimental group by using the number of moles of the effective water per unit mass at the start of foaming without the PBA, $a_{H_{2}O}$, in combination with Equations (10)–(16). The effective rates of different PBAs, $r_{ePBA}$ and $r_{eT}$, are shown in Figure 8 and Figure 9.

It can be seen from Figure 8 that the effectiveness of different PBAs under the same experimental conditions varies greatly, and the overall average effective rate of the PBA relative to the total moles of PBA used varies from approximately 50% to almost 90%. As a second-generation foaming agent, HCFC-141b has the lowest overall average effective rate, while as a third-generation foaming agent, HFC-245fa has the highest overall average efficiency. The fourth-generation blowing agents HFCO-1233zd(E) and HFO-1336mzzZ are in the middle position, and the two themselves are also significantly different.

In all experimental groups, the relationship between the effective rate of the PBA, $r_{ePBA}$, and the initial mass ratio of the PBA to other blending materials in PURF, w, showed a tendency to first decrease and then gradually stabilize or slightly increase. This shows that different PBAs in the foamed material would have the same influencing factors with the change in the addition amount. As the PBA content increases, the number of PBA molecules in the foamed material gradually increases. The influence of the interaction of the PBA molecules among themselves and with other component molecules in the foamed material increases during the heat gasification process. When the PBA content is low, the heat absorbed in the foaming system is lower, and the heating rate of the foaming system is faster. The internal temperature of the foam when it stops expanding during polyurethane foaming, $T_{F}$, is higher. For the PBA, the corresponding gasification conditions are reached earlier, and it is easier to continue gasification. More effective PBA molecules are then produced to expand the foam, and their efficiency, $r_{ePBA}$, is higher. As the PBA content increases, the heat absorbed in the foaming system increases, and the heating rate of the foaming system slows down. The internal temperature of the foam when it stops expanding during polyurethane foaming, $T_{F}$, decreases. These conditions are detrimental to the continuous gasification of the PBA. At the same time, however, due to the enhancement of PBA content, the interaction of the PBA molecules among themselves and with other component molecules in the foamed material gradually strengthens. The absolute number of molecules whose gasification produces effective effects under the same conditions increases accordingly. This condition has a favorable effect on PBA gasification. Under the combined influence of these two effects, the effective rate of the PBA first decreased with increasing w and then gradually stabilized or slightly increased. This indicates that when w was less than 9.05 wt%, the influence of the system temperature dominated, and when w was greater than 9.05 wt%, the interaction of the PBA molecules among themselves and with other component molecules in the foamed material dominated.

**Figure 10:**
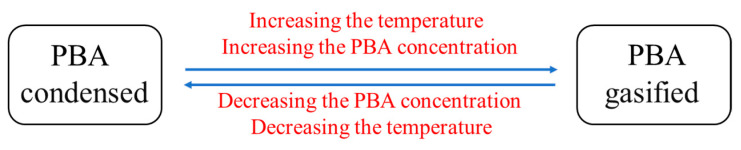
PBA gasification and condensation balance diagram.

From another point of view, this result can also be seen as the equilibrium between PBA gasification and condensation. The final state is that the number of molecules escaping from the foam backbone and being gasified by the PBA is equal to the number of molecules coagulating and dissolving into the foam skeleton. The two reach a dynamic equilibrium, and the foam stops growing. The interaction relationship is shown in Figure 10. Increasing the temperature at the same concentration of PBA or increasing the PBA concentration under the same temperature conditions will shift the equilibrium to the right, and vice versa.

Figure 9 shows that the relationship between the effective rate of the PBA relative to the total mass of materials used, $r_{eT}$ and w, exhibits a good linear trend. This indicates that for a given PURF system using a specific PBA, the ratio of $r_{eT}$ to w is almost constant when the PBA concentration, w, varies in the range of 3.02 wt% to 15.08 wt%. This relationship can be expressed as $r_{eT}=kw$, where k is a variable related to the PBA’s own attributes. In the same PURF system, different PBAs have different k values. The larger the k value, the higher the true efficiency of the PBA.

From the above results, it can be seen that the properties of the PBA itself determine the overall efficiency, while the balance between the gasification and condensation processes of the PBA further leads to a regular change in efficiency with respect to w around the overall average level.

## 5. Conclusions

A complete methodology is developed in the study for measuring the effective rate of a PBA, $r_{ePBA}$, taking into account the properties of the PBA after gasification as a real gas rather than as an ideal gas. This work solves the problem that the effective rate of the PBA could not be directly measured or calculated in previous studies. The effectiveness of different PBAs under the same experimental conditions varies greatly, and the overall average effectiveness rate varies from approximately 50% to almost 90%. In this study, the overall average effective rate of the PBA relative to the total moles of PBA used, $r_{ePBA}$, are as follows in descending order: HFC-245fa, HFO-1336mzzZ, HFC-365mfc, HFCO-1233zd(E), and HCFC-141b.

As the PBA content increases, the density of the polyurethane material after mixing, $\rho_{m}$, increases, and the pressure corresponding to the foam when it stops expanding, $P_{F}$, and the internal temperature corresponding to the foam when it stops expanding, $T_{F}$, both show a downward trend. The factors influencing the three are the density of the PBA, the change in the viscous resistance during foam growth, and the heat absorbed by the PBA per unit mass during the change, respectively. For a given PURF system using a specific PBA, the ratio of the effective rate of the PBA relative to the total mass of materials used, $r_{eT}$, to the PBA concentration, w, is almost constant when w varies in the range of 3.02 wt% to 15.08 wt%. In all experimental groups, the relationship of the effective rate of the PBA relative to the total moles of PBA used, $r_{ePBA}$ and w, show a tendency to first decrease and then gradually stabilize or slightly increase. This trend is determined by the final state when the PBA gasification and condensation reach equilibrium.

In summary, the properties of the PBA itself determine the overall efficiency, while the balance between the gasification and condensation processes of the PBA further leads to a regular change in efficiency with respect to w around the overall average level.

## Figures and Tables

**Table 1:** The relevant properties associated with each generation of the PBA system.

Generation	PBA	Density/g·cm^−3^	Boiling Point/°C	Molecular Weight/g·mol^−1^	ODP	GWP
Second	HCFC-141b	1.24	32	117	0.11	630
Third	HFC-245fa	1.32	15	134	0	1030
Third	HFC-365mfc	1.26	40	148	0	840
Fourth	HFCO-1233zd(E)	1.30	19	131	0	1
Fourth	HFO-1336mzzZ	1.36	33	164	0	2

**Table 2:** Recipes for Group 1 and Group 2 foaming.

Side	Ingredient	Function	Group 1 Weight/g	Group 2 Weight/g
A-side	R4110A	Polyol	100	100
	DMCHA	Catalyst	1.2	1.2
	PC41	Catalyst	0.6	0.6
	DBTDL	Catalyst	0.3	0.3
	H_2_O	Chemical blowing agent	0.5	0.5
	BL-8950	Surfactant	1	1
	TCPP	Fire Retardant	20	20
	PBA (see Table 3)	PBA	—	see Table 3
B-side	PM-200	Polymeric diphenylmethane diisocyanate	208	208

**Table 3:** PBA loading in the foaming recipes for Group 2.

PBA	Weight/g				
	1	2	3	4	5
HFC-245fa	10	20	30	40	50
HFC-365mfc	10	20	30	40	50
HCFC-141b	10	20	30	40	50
HFO-1336mzzZ	10	20	30	40	50
HFCO-1233zd(E)	10	20	30	40	50

## Data Availability

Data is contained within the article.

## Nomenclature

$a_{H_{2}O}$	The number of moles of effective water per unit mass at the start of foaming without PBA, mol·g^−1^
k	The ratio of $r_{eT}$ to w
$m_{a}$	Mass of water added to the mark on the beaker after foaming, g
$m_{b}$	Total material mass at the start of foaming, g
$m_{c}$	Total material mass without the PBA when foaming starts, g
$m_{c_{0}}$	Total mass at the start of foaming in the PBA blank group, g
$m_{f}$	Total material mass after foaming, g
$m_{t}$	The mass of water added to the empty beaker at the scale mark, g
$m_{A}$	Mass of A-side used, g
$m_{B}$	Mass of B-side used, g
$m_{ePBA}$	Mass of the effective PBA, g
$M_{PBA}$	Molar mass of PBA, g·mol^−1^
$n_{{aH}_{2}O}$	Number of moles of effective water in the material total mass at the start of foaming, mol
$n_{ePBA}$	Number of moles of effective PBA, mol
P	Pressure, Pa
$P_{c}$	Critical pressure, Pa
$P_{F}$	Pressure inside the foam when it stops expanding, Pa
$r_{ePBA}$	The effective rate of the PBA relative to the total moles of PBA used, %
$r_{eT}$	The effective rate of the PBA relative to the total mass of materials used, wt%
R	Molar gas constant, J·mol^−1^·K^−1^
$R_{AB}$	Mass ratio of B-side to A-side, wt%
T	Temperature, K
$T_{c}$	Critical temperature, K
$T_{r}$	Contrast temperature
$T_{w}$	Water temperature, K
$T_{F}$	Temperature inside the foam when it stops expanding, K
$V_{A}$	Volume of A-side, cm^3^
$V_{B}$	Volume of B -side, cm^3^
$V_{c}$	Volume of the beaker below the mark, cm^3^
$V_{n}$	Molar volume of real gas, m^3^·mol^−1^
$V_{P}$	Volume of foam actually increased, cm^3^
$V_{{CO}_{2}}$	The part of the volume contributed by the CO_2_ in the foam, cm^3^
$V_{P_{0}}$	Actual increased volume in the PBA blank group, cm^3^
$V_{PBA}$	The part of the volume contributed by the PBA, cm^3^
w	The initial mass content of the PBA in PU materials, wt%
Z	Compressibility factor
$Z_{{CO}_{2}}$	The compression coefficient of CO_2_
$Z_{PBA}$	The compression coefficient of the PBA at the corresponding temperature and pressure
$\rho_{m}$	The density of polyurethane material after mixing, g·cm^−3^
$\rho_{w}$	The density of water, g·cm^−3^
$\rho_{A}$	The density of A-side, g·cm^−3^
$\rho_{B}$	The density of B-side, g·cm^−3^
ω	Acentric factor

## References

[1] Zem A.M. (2022). Bio-Based Rigid Polyurethane Foams Modified with Phosphorus Flame Retardants. Polymers.

[2] Santiago-Calvo M. (2022). Characterization and Properties of Water-Blown Rigid Polyurethane Foams Reinforced with Silane-Modified Nanosepiolites Functionalized with Graphite. Materials.

[3] Jerzak W. (2021). Fly Ash as an Eco-Friendly Filler for Rigid Polyurethane Foams Modification. Materials.

[4] Coccia F., Gryshchuk L., Moimare P., Bossa F.D.L., Santillo C., Barak-Kulbak E., Verdolotti L., Boggioni L., Lama G.C. (2021). Chemically Functionalized Cellulose Nanocrystals as Reactive Filler in Bio-Based Polyurethane Foams. Polymers.

[5] Wang H.Z. (2016). Effect of the Material Temperature on the Foaming Time of Rigid Polyurethane Systems. Polyurethane Ind..

[6] Steed J.M., Owens A.J., Miller C., Filkin D.L., Jesson J.P. (1982). Two-Dimensional Modelling of Potential Ozone Perturbation by Chlorofluorocarbons. Nature.

[7] Loh G., Creazzo J.A., Robin M.L. (2010). Development Programme Update for Low GWP Foam Expansion Agent. Polyurethanes Mag. Int. PU Mag..

[8] Wang H.Z., Lin L., Liu Y.S. (2023). Eco-Friendly Physical Blowing Agent Mass Loss of Bio-Based Polyurethane Rigid Foam Materials. Int. J. Miner. Metall. Mater..

[9] Wang S., Guo Z., Han X., Xu X., Wang Q., Deng S., Chen G. (2019). Experimental Evaluation on Low Global Warming Potential HFO-1336mzz-Z as an Alternative to HCFC-123 and HFC-245fa. J. Therm. Sci. Eng. Appl..

[10] Wysong E., Lee J., Hitchens B. (2018). Opteon 1100: Continued Advancements in Spray Polyurethane Foam Applications. Polyurethanes Mag. Int. PU Mag..

[11] Tsai W.T. (2005). An Overview of Environmental Hazards and Exposure Risk of Hydrofluorocarbons (HFCs). Chemosphere.

[12] Rojas A.J., Marciano J.H., Williams R.J. (1982). Rigid Polyurethane Foams: A Model of the Foaming Process. Polym. Eng. Sci..

[13] Baser S.A., Khakhar D.V. (1994). Modeling of the Dynamics of R-11 Blown Polyurethane Foam Formation. Polym. Eng. Sci..

[14] Baser S.A., Khakhar D.V. (1994). Modeling of the Dynamics of Water and R-11 Blown Polyurethane Foam Formation. Polym. Eng. Sci..

[15] Tesser R., Di S.M., Sclafani A., Santacesaria E. (2004). Modeling of Polyurethane Foam Formation. J. Appl. Polym. Sci..

[16] Harikrishnan G., Khakhar D.V. (2010). Modeling the Dynamics of Reactive Foaming and Film Thinning in Polyurethane Foams. AIChE J..

[17] Suppes G.J., Hsieh F., Tekeei A., Ghoreishi R., Zhao Y., Shen L. Simulation of Urethane Reaction for Both Analytical Methods and Formulation Development. Proceedings of the Polyurethanes Technical Conference.

[18] Zhao Y., Gordon M.J., Tekeei A., Hsieh F.H., Suppes G.J. (2013). Modeling Reaction Kinetics of Rigid Polyurethane Foaming Process. J. Appl. Polym. Sci..

[19] Gandhi K., Kumar R., Niyogi D. (1999). Water Blown Free Rise Polyurethane Foams. Polym. Eng. Sci..

[20] Modesti M., Simioni F., Adriani V. (2000). Chemical and Physical Blowing Agents in Structural Polyurethane Foams: Simulation and Characterization. Polym. Eng. Sci..

[21] Raimbault C., Laure P., Francois G., Boyer S., Vincent M., Choquart F., Agassant J. (2021). Foaming Parameter Identification of Polyurethane Using FOAMAT (R) Device. Polym. Eng. Sci..

[22] Al-Moameri H.H., Hassan G., Jaber B. (2019). Simulation Physical and Chemical Blowing Agents for Polyurethane Foam Production. IOP Conf. Series. Mater. Sci. Eng..

[23] Shen L., Zhao Y., Tekeei A. (2014). Density Modeling of Polyurethane Box Foam. Polym. Eng. Sci..

[24] Wang W.L., Qian Q.H. (2001). Curing and Temperature of Polyurethane Reaction Systems. Chem. Prod. Technol..

[25] Lide D.R., Kehiaian H.V. (2020). CRC Handbook of Thermophysical and Thermochemical Data.

[26] Giacomo P. (1982). Equation for the Determination of the Density of Moist Air (1981). Metrologia.

[27] Sonnenschein M.F. (2020). Polyurethanes: Science, Technology, Markets, and Trends.

